# Supplementary material for: High-throughput automated methods for classical and operant conditioning of Drosophila larvae
Source: eLife. 2022 Oct 28;11:e70015. doi: 10.7554/eLife.70015 (PMC9678368; doi:10.7554/eLife.70015)
Supplement: Transparent reporting form [file elife-70015-transrepform1.pdf]

1st Floor
24 Hills Road
Cambridge CB2 1JP, UK

P 01223 855340
W elifesciences.org
T @elife

eLife's transparent reporting form

We encourage authors to provide detailed information *within their submission* to facilitate the interpretation and replication of experiments. Authors can upload supporting documentation to indicate the use of appropriate reporting guidelines for health-related research (see EQUATOR Network), life science research (see the BioSharing Information Resource), or the ARRIVE guidelines for reporting work involving animal research. Where applicable, authors should refer to any relevant reporting standards documents in this form.

If you have any questions, please consult our Journal Policies and/or contact us: editorial@elifesciences.org.

Sample-size estimation

- You should state whether an appropriate sample size was computed when the study was being designed
- You should state the statistical method of sample size computation and any required assumptions
- If no explicit power analysis was used, you should describe how you decided what sample (replicate) size (number) to use

Please outline where this information can be found within the submission (e.g., sections or figure legends), or explain why this information doesn't apply to your submission:

Proof-of-principle optogenetic and thermogenetic stimulation experiments:
Sample sizes for each genotype are shown below the corresponding data in Figure 2, panels d (optogenetics) and e (thermogenetics).

Aversion to fictive Or42b develops after forward-paired trace conditioning:
Sample sizes for the main statistical analysis for each genotype and conditioning protocol are shown below the corresponding data in Figure 3f.

Operant conditioning of bend direction using high-throughput tracker:
Sample sizes for each genotype and bend direction read-out are shown below the corresponding data in Figure 4, panels c-h, and Figure 5, panels a-d.

Replicates

- You should report how often each experiment was performed
- You should include a definition of biological versus technical replication
- The data obtained should be provided and sufficient information should be provided to indicate the number of independent biological and/or technical replicates
- If you encountered any outliers, you should describe how these were handled
- Criteria for exclusion/inclusion of data should be clearly stated
- High-throughput sequence data should be uploaded before submission, with a private link for reviewers provided (these are available from both GEO and ArrayExpress)

Please outline where this information can be found within the submission (e.g., sections or figure legends), or explain why this information doesn't apply to your submission:

eLife Sciences Publications, Ltd is a limited liability non-profit non-stock corporation incorporated in the State of Delaware, USA, with company number 5030732, and is registered in the UK with company number FC030576 and branch number BR015634 at the address 1st Floor, 24 Hills Road, Cambridge CB2 1JP | August 2014

Proof-of-principle optogenetic and thermogenetic stimulation experiments:
Criteria for data exclusion/inclusion is detailed in the Methods and Materials subsection titled, “Verification of optogenetic and thermogenetic stimulation efficiency”.

Aversion to fictive Or42b develops after forward-paired trace conditioning:
Criteria for data exclusion/inclusion is detailed in the Methods and Materials subsection titled, “High-throughput classical conditioning: Data analysis”.

Operant conditioning of bend direction using high-throughput tracker:
Criteria for data exclusion/inclusion is detailed in the Methods and Materials subsection titled, “High-throughput operant conditioning: Data analysis”.

Visual analysis of Ddc-Gal4 expression pattern
The number of confocal imaging stack replicates for each visual analysis are detailed in Figure 4 – Figure supplement 1.

Statistical reporting

- Statistical analysis methods should be described and justified
- Raw data should be presented in figures whenever informative to do so (typically when N per group is less than 10)
- For each experiment, you should identify the statistical tests used, exact values of N, definitions of center, methods of multiple test correction, and dispersion and precision measures (e.g., mean, median, SD, SEM, confidence intervals; and, for the major substantive results, a measure of effect size (e.g., Pearson's r, Cohen's d)
- Report exact p-values wherever possible alongside the summary statistics and 95% confidence intervals. These should be reported for all key questions and not only when the p-value is less than 0.05.

Please outline where this information can be found within the submission (e.g., sections or figure legends), or explain why this information doesn’t apply to your submission:

Proof-of-principle optogenetic and thermogenetic stimulation experiments:
Statistical analysis tests and exact values of N are described and identified in the Figure 2 caption.

Aversion to fictive Or42b develops after forward-paired trace conditioning:
Raw data is shown in Figure 3, panel f. Statistical analysis tests are described and identified in the Figure 3 caption and in the Methods and Materials subsection titled, “High-throughput classical conditioning: Data analysis”.

Operant conditioning of bend direction using high-throughput tracker:
Statistical analysis tests and precision measures are described and identified in the Figure 4 and Figure 4 captions and in the Methods and Materials subsection titled, “High-throughput operant conditioning: Data analysis”. Exact values of N are displayed in Figure 4, panels c-h, and Figure 5, panels a-d.

(For large datasets, or papers with a very large number of statistical tests, you may upload a single table file with tests, Ns, etc., with reference to sections in the manuscript.)

eLife Sciences Publications, Ltd is a limited liability non-profit non-stock corporation incorporated in the State of Delaware, USA, with company number 5030732, and is registered in the UK with company number FC030576 and branch number BR015634 at the address 1st Floor, 24 Hills Road, Cambridge CB2 1JP | August 2014

Group allocation

- Indicate how samples were allocated into experimental groups (in the case of clinical studies, please specify allocation to treatment method); if randomization was used, please also state if restricted randomization was applied
- Indicate if masking was used during group allocation, data collection and/or data analysis

Please outline where this information can be found within the submission (e.g., sections or figure legends), or explain why this information doesn't apply to your submission:

<u>Aversion to fictive Or42b develops after forward-paired trace conditioning:</u>
Allocation of larvae into stimulation groups is described in the Methods and Materials subsection titled, "High-throughput classical conditioning: Experiment procedures".

<u>Operant conditioning of bend direction using high-throughput tracker:</u>
Allocation of larvae into stimulation groups is described in the Methods and Materials subsection titled, "High-throughput operant conditioning: Experiment procedures".

Additional data files ("source data")

- We encourage you to upload relevant additional data files, such as numerical data that are represented as a graph in a figure, or as a summary table
- Where provided, these should be in the most useful format, and they can be uploaded as "Source data" files linked to a main figure or table
- Include model definition files including the full list of parameters used
- Include code used for data analysis (e.g., R, MatLab)
- Avoid stating that data files are "available upon request"

Please indicate the figures or tables for which source data files have been provided:

All raw data is provided for Figures 2-5. (Note that Figure 1 simply describes our methods). This source data is uploaded with the submission.

eLife Sciences Publications, Ltd is a limited liability non-profit non-stock corporation incorporated in the State of Delaware, USA, with company number 5030732, and is registered in the UK with company number FC030576 and branch number BR015634 at the address 1st Floor, 24 Hills Road, Cambridge CB2 1JP | August 2014
